# A Reinforcement Learning–Based Method for Management of Type 1 Diabetes: Exploratory Study

**DOI:** 10.2196/12905

**Published:** 2019-08-28

**Authors:** Mahsa Oroojeni Mohammad Javad, Stephen Olusegun Agboola, Kamal Jethwani, Abe Zeid, Sagar Kamarthi

**Affiliations:** 1 Department of Information Technology and Analytics Kogod School of Business American University Washington, DC United States; 2 Department of Dermatology, Harvard Medical School Boston, MA United States; 3 Partners HealthCare Boston, MA United States; 4 Mechanical and Industrial Engineering Department, College of Engineering, Northeastern University Boston, MA United States

**Keywords:** type 1 diabetes mellitus, T1DM, diabetes treatment, machine learning, reinforcement learning, Q-learning, insulin dose prescription

## Abstract

**Background:**

Type 1 diabetes mellitus (T1DM) is characterized by chronic insulin deficiency and consequent hyperglycemia. Patients with T1DM require long-term exogenous insulin therapy to regulate blood glucose levels and prevent the long-term complications of the disease. Currently, there are no effective algorithms that consider the unique characteristics of T1DM patients to automatically recommend personalized insulin dosage levels.

**Objective:**

The objective of this study was to develop and validate a general reinforcement learning (RL) framework for the personalized treatment of T1DM using clinical data.

**Methods:**

This research presents a model-free data-driven RL algorithm, namely Q-learning, that recommends insulin doses to regulate the blood glucose level of a T1DM patient, considering his or her state defined by glycated hemoglobin (HbA_1c_) levels, body mass index, engagement in physical activity, and alcohol usage. In this approach, the RL agent identifies the different states of the patient by exploring the patient’s responses when he or she is subjected to varying insulin doses. On the basis of the result of a treatment action at time step t, the RL agent receives a numeric reward, positive or negative. The reward is calculated as a function of the difference between the actual blood glucose level achieved in response to the insulin dose and the targeted HbA_1c_ level. The RL agent was trained on 10 years of clinical data of patients treated at the Mass General Hospital.

**Results:**

A total of 87 patients were included in the training set. The mean age of these patients was 53 years, 59% (51/87) were male, 86% (75/87) were white, and 47% (41/87) were married. The performance of the RL agent was evaluated on 60 test cases. RL agent–recommended insulin dosage interval includes the actual dose prescribed by the physician in 53 out of 60 cases (53/60, 88%).

**Conclusions:**

This exploratory study demonstrates that an RL algorithm can be used to recommend personalized insulin doses to achieve adequate glycemic control in patients with T1DM. However, further investigation in a larger sample of patients is needed to confirm these findings.

## Introduction

### Background

According to the 2017 national diabetic statistics report, diabetes was the seventh leading cause of death in 2015 and a major cause of cardiovascular and renal diseases in the United States [1]. The Centers for Disease Control and Prevention reports that the number of Americans with diabetes is predicted to double or triple by 2050. In 2015, 30.3 million people in the United States (9.4% of the population) had diabetes. Of these, about 1.25 million were reported to have type 1 diabetes mellitus (T1DM) [2,3]. In T1DM, the beta cells responsible for producing insulin in the pancreas are deficient because of autoimmune destruction. T1DM patients depend on lifelong insulin therapy, delivered by injection or a pump, for glycemic control. Uncontrolled blood sugar can lead to serious short-term problems, such as hypoglycemia, hyperglycemia, or diabetic ketoacidosis [1,4-6], or chronic problems that can damage blood vessels supplying blood to important end organs, such as the heart, kidneys, eyes, and nerves [7,8]. Management of T1DM and its complications is achieved via pharmacotherapy, exercise, diet, and other lifestyle changes [9,10]. As individual patients have different physiological characteristics, they respond differently to treatments. Therefore, personalized treatment planning is likely to offer a more effective solution to managing glucose level and diabetes complications.

### Literature Review

Some studies analyzed diabetes data and built models to predict blood glucose level [11-13]. Breault et al (2002) applied a classification and regression tree on data from 15,902 patients with diabetes to predict blood glucose level [14]. Yamaguchi et al (2006) used data collected over a period of 150 days from patients with T1DM to predict next-day-morning fasting blood glucose. They considered metabolic rate, food intake, and physical conditions as predictor variables and concluded that the physical conditions were highly correlated with fasting blood glucose [15]. Bellazzi et al (1998) used a combination of structural time series analysis and temporal abstraction for interpreting historic blood glucose level to extract and visualize the trends and daily cycles of blood glucose level [16]. Bellazzi and Abu-Hanna (2009) applied a temporal abstraction and subgroup discovery algorithm for predicting the blood glucose level of diabetes for 2 types of patients: those who self-monitor their blood glucose level at home and those who were admitted to an intensive care unit [17].

Many studies have used computer-based systems, including open-loop and closed-loop control systems, to control the blood glucose levels of patients with diabetes. In the open-loop system, the patient or diabetologist is responsible for decision making regarding administration of each insulin injection [18]. On the other hand, the closed-loop system mimics the function of the pancreas to control blood glucose level [16-18]. A closed-loop system for T1DM includes either a model-free or a model-based method [19], which follows a cycle of steps: blood glucose measurement, insulin demand calculation, and insulin injection [18]. Many researchers attempted to use model-based control techniques to solve problems associated with diabetes [20,21]. Few studies applied a reinforcement learning (RL) algorithm for controlling blood glucose for type 1 diabetes.

Only a few studies have applied model-based RL algorithm for controlling blood glucose levels for type 1 diabetes. Vrabie et al (2018) proposed using RL for obtaining optimal adaptive control algorithms for dynamical systems using the mathematical models [22]. Ngo et al (2018) used an RL-based algorithm for optimal control of blood glucose in patients with type 1 diabetes using simulations on a combination of the minimum model and part of the Hovorka model [23]. Ngo et al (2018) proposed an RL algorithm for automatically calculating the basal and bolus insulin doses for type 1 diabetes patients using simulation on a blood glucose model with Kalman filter [24].

Currently, there are no effective algorithms to automatically control insulin delivery considering the blood glucose level feedback from the patient body. Only a few studies have attempted a data-driven approach to find a solution. Albisser et al (1974) applied a data-driven approach for developing artificial pancreas based on data from only 3 patients [25]. Javad et al (2015) proposed an RL approach for insulin dosage recommendation for patients with T1DM using an insulin pump based on the data from limited number of patients and states [26].

In this study, we use a data-driven approach where an RL agent learns the model from patient data. The main purpose of this paper is to explore an RL-based approach to recommend personalized treatment plan for managing glucose level to prevent diabetes-related complications and improve quality of life in patients with T1DM.

### Overview of Reinforcement Learning

RL discovers a policy to map a situation to an action to maximize a numeric reward, which takes into consideration not only the immediate rewards but also the possible subsequent rewards (delayed rewards) leading to an outcome such as a state where blood glucose is controlled. An RL agent determines which actions lead to the best reward through exploration of state space and exploitation of experience [27,28]. It has been applied successfully in different scientific fields such as robotics and control [29], manufacturing, and combinatorial search problems such as computer games [30,31]. In health care, using medical image and treatment regimen–related information from historical medical data, RL was used for cancer prediction, diagnosis, and prognosis [32,33].

**Figure 1:**
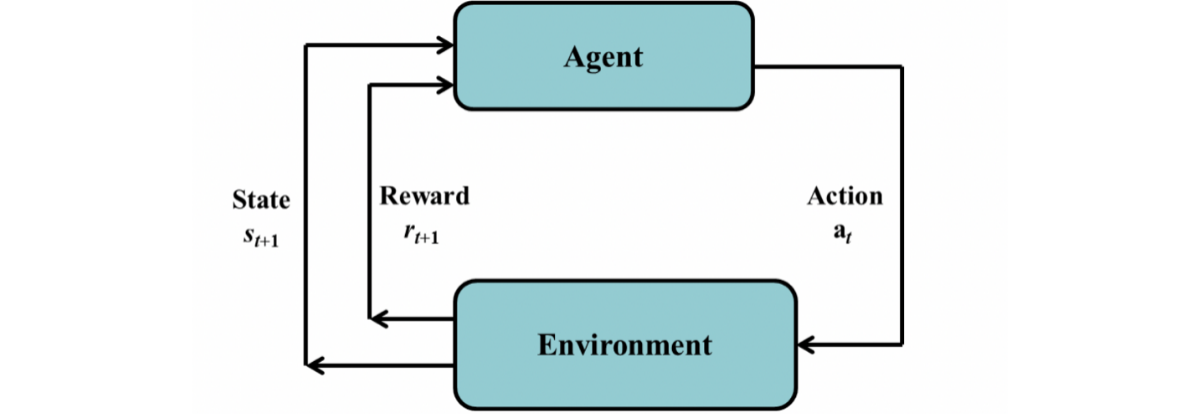
The agent-environment interaction in reinforcement learning.

In RL, the learner or decision maker is called an *agent* (Q-learning in this application; it is described in the Methods section) that interacts with an *environment* (patient with T1DM in this application). Other 4 main subelements of RL include a *policy* (prescription medication level for a given patient condition in this application), a *reward function* (which estimates the reward, either positive or negative, depending on whether or not HbA_1c_ level was controlled), a *value function* (Q-table in this application), and optionally, a *model* of the environment (not used in this application). In this application, let *S* be the set of all possible states of the environment (states of the T1DM patient) and *A* be the set of all possible actions (actions are the insulin levels prescribed to treat the T1DM patient). At each sequence of discrete time steps *t*=0,1,2,3,…, the RL agent receives a representation of the environment’s state *s*_t_∈*S.* Considering available actions when environment is in state *s*_t_, the agent takes an action *a*_t_∈*A*, randomly at the early exploratory learning stage and more rationally exploiting the experience gained through data-driven learning in the advanced learning stage. The RL agent, depending on the consequence of its action at time *t*, receives a numerical reward *r*_t_ and changes the environment to state *s*_t__+1_. Normally, the merit of an action is quantified by the total amount of reward that the RL agent can expect to accumulate in the long run, considering the states that are likely to be visited in the transition. Over a series of learning epochs, the RL agent learns an optimal control policy π^*^: *S* →*A*. At each time step time *t*, the optimal policy π^*^(*s*_t_) maps state *s*_t_ to a right action *a*_t_, that is, *a*_t_= π^*^(*s*_t_). Figure 1 shows the agent-environment (agent-patient) interaction in RL. The optimal control policy is shaped through exploration in the early stages of learning and through experience in the mature stage of learning.

In this study, we apply a data-driven model-free RL method, known as Q-learning, that needs no previous knowledge of the environment to prescribe medication dose to treat T1DM patients considering their current HbA_1c_, body mass index (BMI), activity level, and alcohol usage.

## Methods

This section describes Q-learning as applied to T1DM and its components including parameters that define state space and action space, reward function, training processes, training data, and evaluation function.

### Q-Learning

Q-learning is useful for finding optimal strategies for an environment for which neither the transition function nor the probability distribution of state variables is known [34]. Q-learning works by estimating a set of Q-values, which serves as the role of a value function. In the Q-learning algorithm, Q-values are estimated for each state-action (*s*_t_,*a*_t_) combination. Once the final Q-values are estimated, the only thing that needs to be known is the state of the environment (T1DM patient) *s*_t_ to determine a right action *a*_t_ (insulin dose).

**Figure 2:**
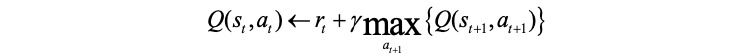
Q-value update function.

At the beginning of the algorithm, Q-values are initiated to an arbitrary real number. Subsequently, at each iteration *t*, for each combination of state *s*_t_∈*S* and action *a*_t_∈*A*, a reward value is calculated by the RL agent. At the core of the algorithm is the iterative process of updating Q-values as a function of the immediate reward *r*_t_ and Q-values of the next state-action pair Q(*s*_t__+1_, *a*_t__+1_). Figure 2 shows Q-value update function.

**Figure 3:**
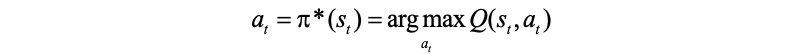
Optimal policy function.

In the above formulation, *γ* is a factor that regulates the influence of the future rewards relative to the current reward. If *γ*=0, the reward only depends on the reward received in the current state; as *γ* approaches 1, the reward is maximized over the long run taking future rewards into consideration [27,28]. Over several iterations of learning, Q-values for state-action pair, Q(*s*_t_, *a*_t_), converge to stable values and the RL agent is considered to have learned the optimal policy π^*^:S→A. At each time step time *t*, given state *s*_t_, the right action *a*_t_ is determined from the formula presented in Figure 3.

### Q-Learning Applied to Type 1 Diabetes Mellitus

In this study, we study a Q-learning algorithm that prescribes medication level to a T1DM patient considering his or her state defined by HbA_1c_, BMI, activity level, and alcohol usage. The data for training Q-learning were obtained from electronic health records (EHRs) of patients admitted to the Mass General Hospital (MGH).

#### Parameters That Define State Space

On the basis of American Diabetes Association report, several factors such as diet, medication adherence, alcohol usage, physical activity, BMI, stress, age, smoking status, and side effects from other medications can change the blood glucose level of diabetes patients [1]. To identify the factors that are crucial for developing an effective machine learning model to personalize diabetes treatment planning, we calculated the correlation coefficient matrix of potential variables recorded in the EHR and observed that only BMI, activity level, and alcohol usage were strongly correlated with the blood glucose level measured in terms of HbA_1c_; other potential variables, such as age and smoking status, did not show significant correlation coefficients. Therefore, in this study, we defined a patient’s state by the 4 factors that influence the patient’s future HbA_1c_: current HbA_1c_, BMI, activity level, and alcohol usage.

We denote the set of HbA_1c_ states at epoch *t* by 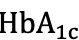
_t_={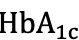
_at_|*a*=1,2,3}, the set of BMI levels by BMI_t_={BMI_bt_| *b*=1,…,17}, the set of activity levels by activity_level_t_={activity_level_ct_| *c*=1,2}; and the set of alcohol usage levels by alcohol_usage_t_={alcohol_usage_dt_| *d*=1,2,3}. Table 1 presents the levels for HbA_1c_, BMI, activity level, and alcohol usage. The set of health states of a T1DM patient at epoch *t* is defined by *s*_t_=(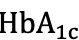
_t_, BMI_t_, activity_level_t_, alcohol_usage_t_).

**Table 1:** Definitions of levels for glycated hemoglobin, body mass index, activity level, and alcohol usage.

Variable	Level 1	Level 2	Level 3	Levels 4 to 16	Level 17
Glycated hemoglobin	≤7: glucose level is well controlled	(7,9]: glucose level is moderately controlled	>9: glucose level is poorly controlled	NA^a^	NA
Body mass index distribution	[18.5,19)	[19,20)	[20,21)	[21,22) to [33,34)	(34,35]
Activity level	Active—engages in physical activity ≥2 times per week	Nonactive—engages in physical activity <2 times a week	NA	NA	NA
Alcohol usage	Mild to no alcohol consumption—consumption of alcohol <2 times a week	Moderate to high alcohol consumption—consumption of alcohol ≥2 times per week	Heavy consumption—consumption of alcohol few times a day	NA	NA

^a^Not applicable.

#### Parameters That Define Action Space

Insulin is the mainstay of T1DM treatment and mostly administered through injections. The type of insulin that a T1DM patient needs depends on the severity of insulin depletion. There are different types of insulin used to treat T1DM. Normally, these insulin supplements are classified as short, rapid, intermediate, or long-acting. In this exploratory research, we focus only on the prescription of the most commonly prescribed long-acting insulin, that is, insulin glargine, which goes by the common brand name Lantus.

Lantus is usually injected once per day at the same time each day. Once injected, Lantus works for about 24 hours. This is similar to the action of insulin normally produced by the pancreas to keep a patient’s blood sugar under control throughout the patient’s daily routine. Adding rapid-acting insulin to the long-acting background insulin prevents increasing a patient’s blood glucose right after eating a meal [7]. In the proposed Q-learning algorithm, actions represent the Lantus medication dosage levels recommended to the patients. Possible actions are coded based on 6 Lantus dosage ranges: *a*_1__t_=[6,15), *a*_2__t_=[15,20), *a*_3__t_=[20,30), *a*_4__t_=[30,40), *a*_5__t_=[40,50), and *a*_6__t_=[50,100]; these levels are referred to as Action 1, Action 2, …, Action 6, respectively. The set of possible actions at epoch *t* is denoted by *a*_t_={ *a*_kt_| *k*=1,2,…,6}, in other words, *a*_t_={Action 1,Action 2,…,Action 6}. Actions are taken at a discrete decision epoch indexed by *t=* 1,2,..., *T*, where epoch *t* represents the time of the patient’s visit to physician’s office to get checkup and Lantus prescription. The patient’s visits (approximately every 3 months) to their physician over 10 years are treated as decision epochs.

#### Reward Function

**Figure 4:**
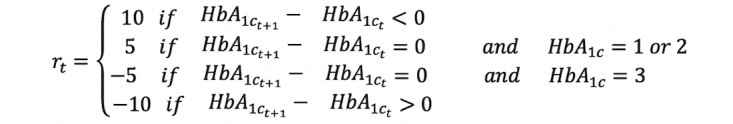
Reward function.

In the proposed algorithm, the RL agent receives reward at each state comparable with the change in the state of HbA_1c_. At the beginning, the patient is in state *s*_1_ and takes treatment action *a*_1_; as a result, the agent receives reward *r*_1_ and the patient moves on to state *s*_2_; then the patient takes treatment *a*_2_, the agent receives reward *r*_2_, and the patient reaches state *s*_3_; and the procedure continues in this fashion. From a series of data-driven experiences, the RL agent learns the right action *a*_t_ (prescription of right Lantus dose) for a given patient state *s*_t_. Figure 4 shows the reward function for the Q-learning algorithm.

#### Training Processes

**Figure 5:**
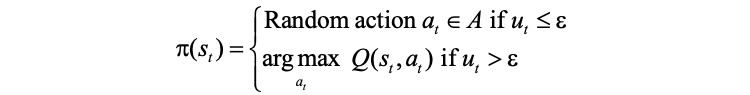
Random action selection function.

In the training process, the Q-learning agent in this algorithm tries to learn the optimal treatment policy from the patient’s historical data in the EHR. At each iteration, the agent updates a table of Q-values for each combination of state and action. For example, each experience cycle (*s*_t_, *a*_t_, *s*_t__+1_, *r*_t_) updates the value of Q(*s*_t_, *a*_t_) according to the Equation 1. In this implementation, ε-greedy policy is applied for taking actions during the training process. Implementing ε-greedy policy helps the algorithm visit and explore different states by choosing random actions with small probability ε, instead of always taking experience-driven promising actions all the time. In this method, at each time step *t*, the algorithm selects a random action with a fixed probability, ε, based on the following formulation. Figure 5 shows the random action selection function, where 0≤ *u*_t_≤1 is a uniform random number drawn at each time step *t* [23,24].

#### Training Data

RL algorithm was trained and tested on the clinical data obtained from the MGH. The study was approved by the Partners Human Research Committee, the institutional review board that grants approval for such studies. In the dataset, most of the patients used Lantus compared with other types of insulin. So, this exploratory research focuses on only Lantus treatment planning for T1DM. Medical records of 87 T1DM patients enrolled at MGH from 2003 to 2013 were included in the training set. Only the patients who had complete data necessary for training the Q-learning agent were included in this analysis. Medical record data for each patient’s visits over a 10-year period were collected and processed for analyses. At each clinical encounter, HbA_1c_, BMI, activity level, alcohol usage status, and Lantus medication dose were recorded. Table 2 shows a sample of patient data collected from each visit. In addition, we validated the trained Q-learning agent performance on another dataset with 60 MGH patients for whom complete data were available.

#### Evaluation Function

**Figure 6:**
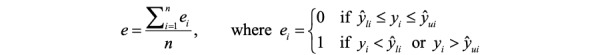
Error function.

Consider that (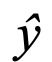
*_li_*, 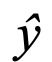
*_ui_*) is the Lantus dose interval recommend by the RL agent for test case *I*, and *y*_i_ is the actual Lantus dose prescribed by the patient’s physician, and there are *n* number of cases in the validation set. The following equation was used for calculating the average error of RL agent predications. Figure 6 shows error function.

**Table 2:** Tracking the patients’ visits.

Visit	HbA_1ct_	Body_mass_index_t_	Activity_level_t_	Alcohol_usage_t_	Lantus_dose_t_
1	8.1	21.4	1	1	20
2	9.1	24	1	1	22
3	8	22	1	1	21

## Results

The average age of the study population was 53 years, 59% of the patients were male, 86% were white, and 47% were married. Table 3 shows demographics characteristics of patients included in the training data.

Table 4 shows demographics characteristics of patients included in the testing data. Table 5 presents the results of Q-learning algorithm for 60 test cases. For the 60 test patients, on average, in 53 out of 60 cases (88%) the physician-prescribed Lantus dose was within the dose interval recommended by the Q-learning algorithm.

**Table 3:** Summary of training data of patients (N=87).

Patient characteristics		Statistics^a^
**Age (years)**		
	Mean (SD)	52.9 (15.7)
	Median	54
**Race distribution, n (%)**		
	White	75 (86)
	Hispanic or Latino	7 (8)
	Black	3 (3)
	Asian	1 (1)
	Not recorded	1 (1)
**Marital status, n (%)**		
	Married or partnered	41 (47)
	Single or widow	33 (38)
	Divorced or separated	12 (14)
	Widowed	1 (1)
**Gender, n (%)**		
	Male	51 (59)
	Female	36 (41)

^a^Due to rounding, the sum of the percentages shown is not 100.

**Table 4:** Summary of test data of patients (N=60).

Patient characteristics		Statistics
**Age (years)**		
	Mean (SD)	50.4 (15.8)
	Median	52
**Race distribution, n (%^a^)**		
	White	53 (88)
	Hispanic or Latino	5 (8)
	Not recorded	2 (3)
**Marital status, n (%^a^)**		
	Married or partnered	32 (53)
	Single or widow	22 (36)
	Divorced or separated	6 (10)
**Gender, n (%)**		
	Female	34 (57)
	Male	26 (43)

^a^Due to rounding, the sum of the percentages shown is not 100.

**Table 5:** Test results.

Test number	Hemoglobin A_1c_ level	Body mass index level	Activity level	Alcohol usage	Actual Lantus Units dosage prescribed	Reinforcement learning agent–recommended Lantus Units dose interval	Comparison of actual Lantus dose with reinforcement learning agent–recommended Lantus dose interval
1	1	7	1	1	6	[6,15)	match
2	1	2	1	1	14	[6,15)	match
3	2	5	1	1	12	[6,15)	match
4	2	7	1	1	20	[6,15)	not match
5	2	4	1	1	14	[6,15)	match
6	2	5	1	1	10	[6,15)	match
7	2	6	1	1	12	[6,15)	match
8	2	8	1	1	20	[20,30)	match
9	2	4	1	1	20	[20,30)	match
10	2	11	1	1	25	[20,30)	match
11	2	10	1	1	25	[20,30)	match
12	2	8	1	1	13	[20,30)	not match
13	2	6	1	1	10	[6,15)	match
14	2	2	1	1	11	[6,15)	match
15	2	4	1	1	12	[6,15)	match
16	2	5	1	1	13	[6,15)	match
17	2	7	1	1	22	[6,15)	not match
18	2	4	1	1	8	[6,15)	match
19	2	4	1	1	6	[6,15)	match
20	2	4	1	1	9	[6,15)	match
21	2	5	1	1	10	[6,15)	match
22	2	5	1	1	14	[6,15)	match
23	2	8	1	2	15	[6,15)	match
24	2	14	1	2	20	[20,30)	match
25	2	8	1	2	18	[6,15)	not match
26	2	5	1	2	14	[6,15)	match
27	2	5	1	2	14	[6,15)	match
28	2	5	1	2	8	[6,15)	match
29	2	8	1	2	15	[6,15)	match
30	2	5	1	2	11	[6,15)	match
31	2	5	1	2	10	[6,15)	match
32	2	4	1	2	9	[6,15)	match
33	2	6	1	3	20	[20,30)	match
34	2	5	1	3	20	[20,30)	match
35	2	5	1	3	20	[20,30)	match
36	1	6	1	3	20	[15,20)	match
37	3	12	2	1	46	[30,40)	not match
38	1	5	2	1	15	[15,20)	match
39	1	7	2	1	20	[15,20)	match
40	1	5	2	1	15	[15,20)	match
41	2	8	2	1	25	[20,30)	match
42	2	10	2	1	30	[20,30)	match
43	3	13	2	1	50	[30,40)	not match
44	2	8	2	1	21	[20,30)	match
45	2	6	2	1	20	[20,30)	match
46	2	8	2	1	28	[20,30)	match
47	2	6	2	1	20	[20,30)	match
48	2	7	2	1	20	[15,20)	match
49	3	12	2	1	50	[50,100]	match
50	3	17	2	1	70	[50,100]	match
51	3	17	2	1	80	[50,100]	match
52	2	8	2	1	25	[20,30)	match
53	2	6	2	1	30	[20,30)	match
54	3	12	2	1	50	[30,40)	not match
55	3	11	2	1	36	[30,40)	match
56	3	11	2	1	38	[30,40)	match
57	2	8	2	1	21	[20,30)	match
58	2	9	2	1	23	[20,30)	match
59	2	9	2	1	23	[20,30)	match
60	2	7	2	1	20	[20,30)	match

## Discussion

### Principal Findings

Alcohol usage, physical activity, BMI, stress, and HbA_1c_ level are crucial for developing effective models to personalize diabetes treatment planning [1]. In this study, a Q-learning agent that predicts personalized insulin dosages was formulated, trained, and tested considering patients’ current HbA_1c_, BMI, activity level, alcohol usage to define the patient state at epoch *t*: *s*_t_={
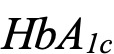
_t_, *BMI*_t_, *activity_level*_t_*, alcohol_usage*_t_}. In other words, a patient can be in any of the 306 possible states (number of HbA_1c_ states*number of BMI states*number of activity level states*number of alcohol usage status states=3 × 17 × 2 × 3). Each of these combinations represents a state. For example, if the patient is in state *s*_t_, the dosage recommendation *a*_t_, appropriate to state s_t_, is suggested by Q-learning agent for that patient. Q-learning agent–recommended Lantus dose interval includes the actual prescription dose in 88% of the cases.

### Limitations

This research has several limitations. We did not include other important lifestyle information about patients’ diet, stress, and medication adherence. These are well-known factors that influence blood glucose levels but are infrequently documented in the medical records. We suggest considering these factors in future research for developing more effective blood glucose control. Another important limitation is the small training dataset. The main constraint to evaluating the model in a larger cohort of patients was the time it took to clean and extract these important but poorly documented factors. With adequate funding, we can apply more sophisticated natural language processing techniques to capture data from unstructured text or note from a larger sample of patients. Yet another factor is the limited generalizability of the study findings. Study data were from patients in a large academic medical center that has a diabetes center and access to other supportive lifestyle change programs that may not be available in community health centers. The fact that only 1 type of insulin (Lantus) was included broadly limits the application of this study. However, as a proof of concept, we demonstrated that this concept could potentially be used for other insulin regimen as well.

### Comparison With Previous Studies

Although in recent years, we have seen increased interest in applying machine learning methodologies in the study of personalize diabetes treatment planning, this study is the first of its kind that aims at finding the best insulin dosage for the T1DM for several reasons. First, this study involved the use of crucial factors including alcohol usage, physical activity, BMI, and HbA_1c_ level for finding the best insulin dosage for patients with type 1 diabetes. None of the earlier studies in the literature considered all of these important factors for developing effective models to personalize diabetes treatment planning. Second, 2 patients with the same BMI and HbA_1c_ but different alcohol usage and activity level need different insulin dosages for managing their blood glucose level. Considering only BMI and HbA_1c_ for insulin dosage recommendation may lead to suggesting the same dose of medication to patients with different insulin dosage needs. Finally, this study involved the use of a larger clinical dataset compared with other datasets used in other studies concerned with managing blood glucose level. Data gathered from clinical settings have an important and complementary role in the research outcomes. The suggested model-based approaches in the literature used mathematical models for simulating the function of pancreas. These model-based approaches did not consider patient’s alcohol usage and physical activity level for the insulin dosage recommendation.

Yasini et al (2003) applied an agent-based simulation for managing blood glucose of patients with diabetes based only on blood glucose levels [19]. For each state of glucose level, their algorithm provided only 1 insulin dosage recommendation without considering the patient’s BMI, activity level, or alcohol usage. Our proposed algorithm provides more precise insulin dosage recommendation considering the patient’s current HbA_1c_, BMI, activity level, or alcohol usage. Vrabie et al (2018) and 2 studies by Ngo et al (2018) applied a model-based RL algorithm for controlling blood glucose for type 1 Diabetes [22-24]. We used a data-driven approach and considered the blood glucose level feedback from the patient body for training the Q-learning algorithm. In addition, our proposed Q-learning algorithm considers not only the blood glucose of the patient for the insulin dosage recommendation but also the patient’s current HbA_1c_, BMI, activity level, and alcohol usage. Javad et al (2015) applied data-driven approach on the limited number of patients and small dimension of problem with only 13 states for insulin dosage recommendation of type 1 diabetes, without testing the results [26]. Our proposed algorithm provides more precise insulin dosage recommendation based on the 306 possible patient states, and the results have been validated. RL algorithm was trained on the clinical data obtained from 87 T1DM patients enrolled at MGH from 2003 to 2013. Furthermore, the performance of the RL agent was evaluated on 60 test cases.

### Conclusions

Effective decision making about correct insulin dose may delay or prevent diabetes complications, such as heart attack, kidney disease, blindness, and amputation [2]. Study findings suggest that physicians may be able to use a Q-learning agent that considers patients’ BMI, activity level, alcohol usage status, and current HbA_1c_ level to recommend insulin doses. This machine learning model may help improve the timeliness of achieving an effective treatment dose rather than multiple dosage trials based on clinical acumen alone. In addition to improving treatment efficacy time, this has the potential to reduce patient stress (less clinic visits), reduce health care costs, and improve overall quality of life. Future research could extend this proof-of-concept Q-learning model to include other types of insulin and other types of diabetes medications and other state variables. The performance of the Q-learning model can be enhanced by considering finer categories and intervals for defining a patient state and action. It may also be worth exploring in patients with type 2 diabetes.

## Abbreviations

BMI: body mass index
EHR: electronic health record
HbA _1c_: glycated hemoglobin
MGH: Mass General Hospital
RL: reinforcement learning
T1DM: type 1 diabetes mellitus
